# Real-Time and Tunable Substrate for Surface Enhanced Raman Spectroscopy by Synthesis of Copper Oxide Nanoparticles via Electrolysis

**DOI:** 10.1038/s41598-017-08199-0

**Published:** 2017-08-10

**Authors:** Behzad Sardari, Meriç Özcan

**Affiliations:** Faculty of Engineering and Natural Sciences, Sabancı University, İstanbul, Turkey

## Abstract

Here we show the capability of copper oxide (CuO) nanoparticles formed on copper (Cu) electrodes by the electrolysis as a real time active substrate for surface enhanced Raman scattering (SERS). We have experimentally found that using just the ultra pure water as the electrolyte and the Cu electrodes, ions are extracted from the copper anode form copper oxide nanoparticles on the anode surface in matter of minutes. Average particle size on the anode reaches to 100 nm in ninety seconds and grows to about 300 nm in five minutes. This anode is used in Raman experiments in real time as the nanoparticles were forming and the maximum enhancement factor (*EF*) of Raman signals were over five orders of magnitude. Other metal electrodes made of brass, zinc (Zn), silver (Ag) and aluminum (Al) were also tried for the anode material for a possible real-time substrate for SERS applications. Experimentally obtained enhancement factors were above five orders of magnitude for brass electrodes like the copper but for the other metals no enhancement is observed. Electron microscope images show the cubic nanoparticle formation on copper and brass electrodes but none in the other metals studied.

## Introduction

Surface Enhanced Raman spectroscopy (SERS) has emerged as a powerful spectroscopy technique to increase the intensity of Raman scattering signals from the analyte of interest. It is generally accepted that the main mechanism of the enhancement is due to the amplification of the light by excitation of localized surface plasmon resonances (LSPR) of the metallic nanoscale features and/or nanoparticles present on the surface where the analyte is put onto^1^. SERS was introduced in the mid-1970s^2–4^, and since then significant research has been done to create and optimize SERS substrates with high density of hot spots in order to obtain the largest enhancement factor (*EF*)^5–8^. Hot-spots are the locations near nanostructures where the local optical field is enhanced, so that Raman scattering signals from the molecules near the hot spots are enhanced 10^4^ to 10^7^ times. Even much higher enhancement is possible with some special substrates enabling single molecule detection. The *EF* is generally written as^9, 10^:

1 $$EF=\frac{{|{E}_{loc}({\omega }_{L})|}^{2}}{{|{E}_{inc}|}^{2}}\times \frac{{|{E}_{loc}({\omega }_{R})|}^{2}}{{|{E}_{inc}|}^{2}},$$

where *E*_*inc*_ is the amplitude of the incident laser light, and *E*_*loc*_(*ω*_*L*_) and *E*_*loc*_(*ω*_*R*_) are the local electric field amplitude at the incident laser frequency and the local electric field amplitude at the scattered Raman signal frequency respectively. In most cases the Raman shift is small enough to justify the approximation: *ω*_*L*_ ≈ *ω*_*R*_, in which case the SERS enhancement is simply proportional to the fourth power of the local field enhancement:

2 $$EF\approx \frac{{|{E}_{loc}({\omega }_{L})|}^{4}}{{|{E}_{inc}({\omega }_{L})|}^{4}}\mathrm{.}$$

Over the years, generally, two different ways of building a SERS active substrate has emerged; coating a substrate with pre-synthesized nanoparticles by techniques such as chemical synthesis, colloidal aggregation etc., which are called as bottom-up methods^1, 11–16^, and fabrication of a nanoparticle template on a substrate by the nanofabrication techniques such as electron beam lithography (EBL), focused ion beam (FIB), scanning probe lithography etc., which are called as top-down methods^17–21^. Synthesis of colloidal nanoparticles is straightforward but it is too time consuming process, however it is appropriate for large scale production. Also, the nanoparticles accumulation is another disadvantage of this kind of process. The patterned substrates have advantages compared to the nanoparticle colloids such as adjusting the inter-particle distance, uniformity in enhancement factor, repeatability, and absence of particle aggregation issues. However, it has disadvantages as it is a time consuming process, more complicated and costly and also needs high tech fabrication facilities.

Recently, some researchers focused on synthesis of colloidal nanoparticles by electrolysis method for applications in the inkjet printing and SERS^22–25^. Synthesis of nanoparticles by electrolysis is a bottom-up method which is accomplished due to reduction and oxidation at the cathode and anode respectively and compared to most of the other techniques this is a simple and economical method. Therefore, electrochemical method is one of the simplest way of producing copper oxide nanoparticles which is discussed in the literature^25–31^. For example, Yuan *et al*.^25^ have described the effect of current and electrolyte on the shape and size of the copper oxide nanostructures. Xu *et al*.^26^ have synthesized leaf-like CuO mesocrystals by the electrochemical method then have discussed their capability as anode materials for lithium ion batteries. Lu *et al*.^27^ have synthesized flower-like microspheres CuO by using sodium hydroxide as the electrolyte solution at room temperature. Toboonsung *et al*.^31^ have synthesized CuO nanorods by an electrochemical dissolution and deposition process then studied the electrode separation and voltage effect etc. on the nanorods morphology and thickness.

Here we present a simple, real-time operating and wavelength tunable copper SERS substrate formed by the electrolysis. There are already widely used electrolysis methods for synthesis of nanoparticles as mentioned earlier, however those studies include metals salts in the electrolyte, and it takes hours to days for nanoparticle production in the solution or on the cathode. There are reports which indicate nanoparticle production on the cathode^31^ and the anode^32^ with distilled water alone but their measurements were done after hours of electrolysis. Therefore it was quite surprising when we have observed cube shaped nanoparticles on the copper anode surface in matter of a few minutes using just the ultra-pure water (UPW) as the electrolyte. Our measurements indicate that the ions are extracted from the Cu anode form cube shaped copper oxide nanoparticles on the anode surface in such a short time, and we show that this anode can be used as a SERS substrate. Initially, the anode is removed from the electrolysis cell and a solution of rhodamine B (RhB) is applied over the surface to perform Raman spectroscopy. We obtained enhancement of Raman signals over five orders of magnitude. Afterwards, the experimental setup was modified to allow real-time Raman measurements of RhB while the electrolysis is ongoing, and we again obtained enhancement over five orders of magnitude within 90 seconds of electrolysis. Similar enhancements were obtained with crystal violet (CV) solutions as well. The proposed method has some key advantages over existing SERS substrates: its not only a real time SERS substrate but also is a very fast, simple and a low cost technique. Furthermore this substrate is tunable in wavelength -albeit only irreversibly and in one direction- as the particle size is increasing as a function of time which implies that plasmon resonance wavelengths are increasing as well. This technique also omits the need for an electrolyte of containing the metal ions of interest for the nanoparticle production as just the deionized or distilled water is enough. This is an important point for the Raman measurements because the electrolyte being simply just the water there will not be an extra background noise added to the spectrum. This technique also allows preparation of a large effective area for SERS enhancement, virtually unlimited area. As long as the current distribution over the anode is uniform which is a trivial arrangement, nanoparticle distribution will have quite homogenous distribution. Other metal electrodes made of brass, zinc (Zn), silver (Ag) and aluminum (Al) also were tried as the anode but except brass none of the other metals showed any enhancement. Also it is confirmed with electron microscope images that there was nanoparticle formation on copper and brass electrodes but none on the other metals studied.

In the following sections experimental setup and the performed SERS experiments are presented. Experimentally observed enhancement factors were more than five orders of magnitude for copper and brass electrodes while for the other metals no enhancement is observed. Later we will discuss the possible mechanism of the nanoparticle production on the anode surface which we think the standard electrode potentials of the electrodes play a key role. Also, surface plasmon resonance simulations of the nanoparticles were performed in accord with the nanoparticle distributions obtained from the SEM images. The simulation results agree with the change of SERS intensity as a function of time since as the nanoparticles grow in size SPR wavelengths shift.

## Experimental Setup and The Results

**Figure 1:**
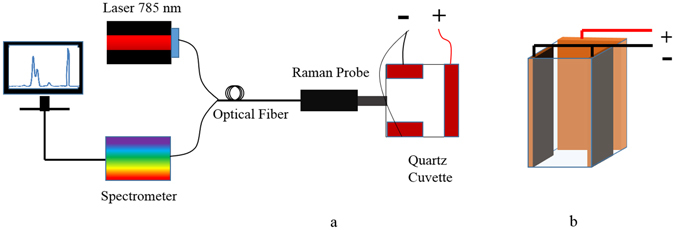
(**a**) Drawing of the experimental setup for preparing real time SERS substrate and recording the Raman spectrum simultaneously. (**b**) Quartz cuvette used as the electrolysis chamber and as a container simultaneously for the solution that the Raman spectrum is of interest. The anode and cathode electrode dimensions are 50 *mm* × 8.5 *mm* × 0.25 *mm* and 50 *mm* × 5.5 *mm* × 0.25 *mm* respectively. The Raman signal was recorded head-on from the anode surface.

Three surface-cleaned copper electrodes have been placed inside a 3 mL quartz cuvette in which two of them were put in parallel, facing each other and electrically connected together while the third one is perpendicular to these electrodes, as illustrated in Fig. 1. The single electrode is used as the anode and the two parallel electrodes are used as the cathode. The reason that the anode and cathode are not located face to face is because we wanted to place the Raman probe facing the anode from the remaining facet of the cuvette.

**Figure 2:**
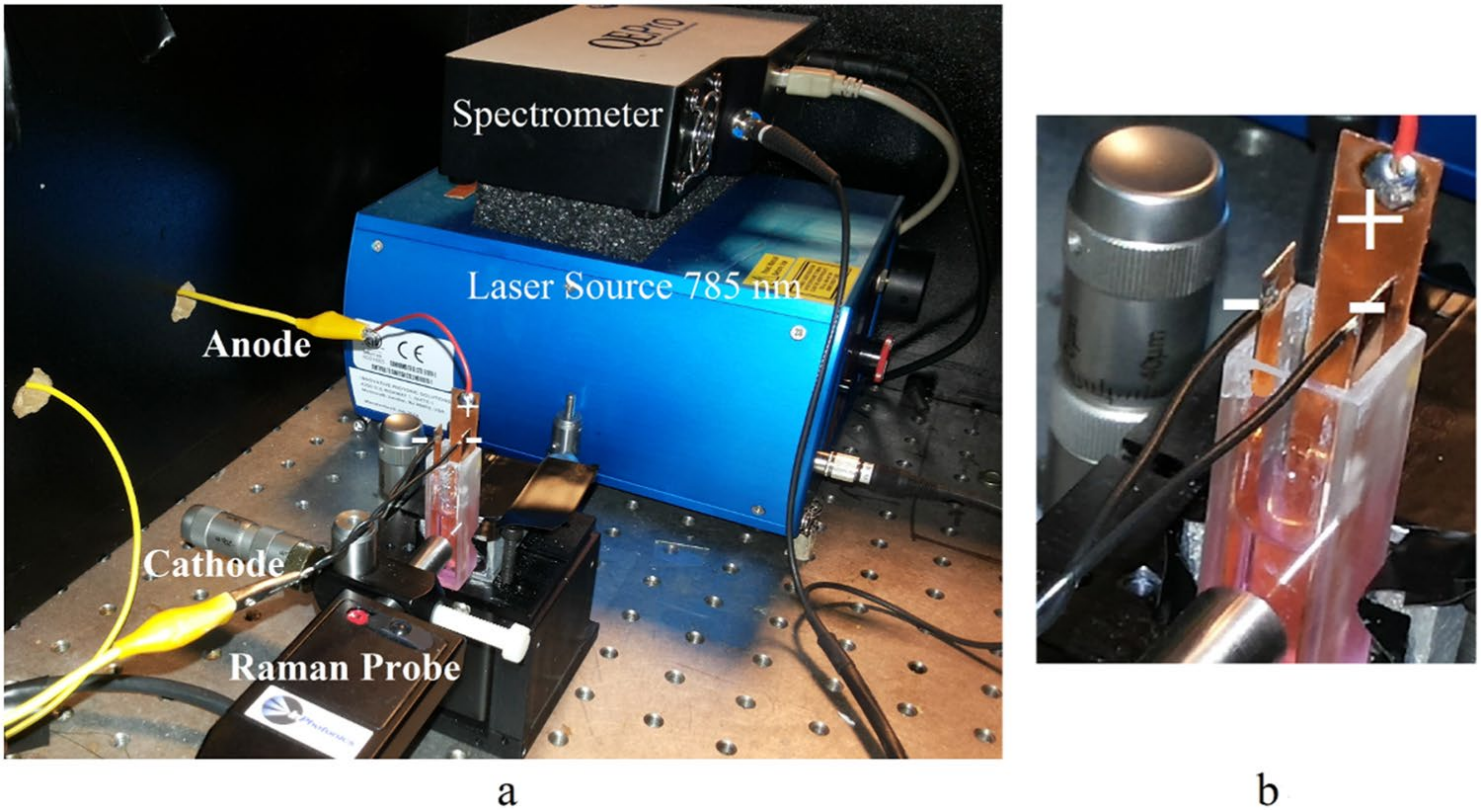
A photograph of the experimental setup for substrate preparation and recording the Raman spectrum. Quartz cuvette and the Raman probe are both set on optical XYZ stages for ease of alignment. Laser light is launched from the Raman probe and the back-scattered light from anode surface propagates back through the Raman probe to the spectrometer.

In order to be able to record Raman spectrum of analytes while the nanoparticles are formed on the anode, the Raman probe is set in front of the quartz cuvette on an XYZ optical stage for ease of alignment. Longitudinal alignment (z-axis) of the probe is necessary for focusing the light source at the anode surface and capturing the back-scattered Raman signal, while lateral positioning is necessary for accessing the different parts of the anode surface. Raman probe is made by InPhotonics Inc., the spectrometer (QEpro) and the 785 *nm* infrared laser source with adjustable power up to 500 *mW* are made by Ocean Optics Inc. Laser light is launched from the Raman probe and the back-scattered light from anode surface propagates back through the Raman probe to the spectrometer. A photograph of the experimental set-up is shown in Fig. 2.

**Figure 3:**
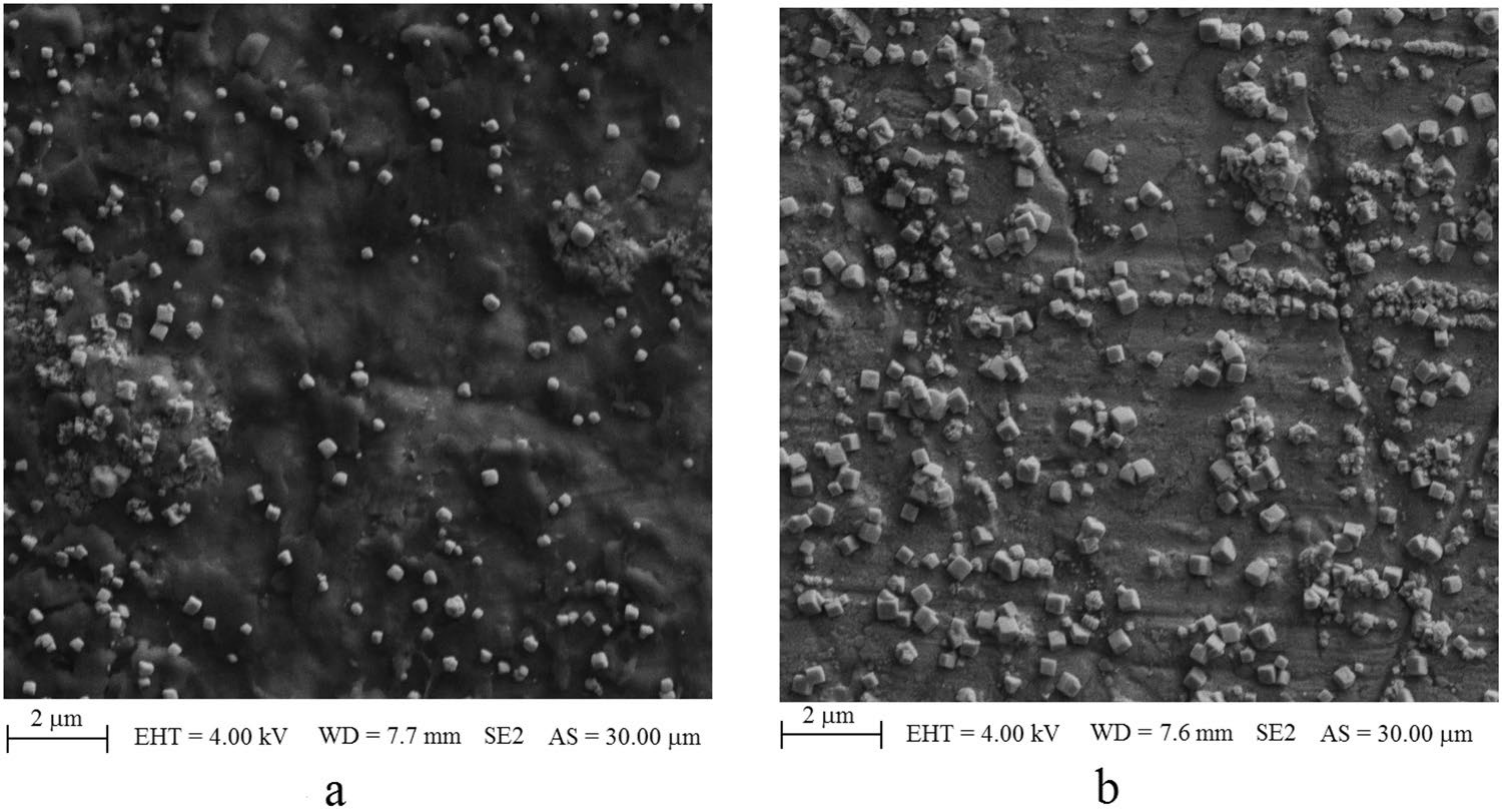
SEM images of two different areas from anode surface after 5 minutes of electrolysis while the container was filled with ultra-pure water.

Initially, the cuvette is filled with ultra-pure water, and just the electrolysis is performed without any Raman measurement. The conductivity of the water was 55 *nS*/*cm*, and the applied voltage between the anode and the cathode was 32 *V*. After five minutes of electrolysis the anode electrode was removed and dried. The captured scanning electron microscope (SEM) images of two different areas from the anode surface after five minutes electrolysis as shown in Fig. 3 confirms the production of cubic nanoparticles with dimensions up to 400 *nm*. It will be explained later that the nanocubes are actually CuO particles.

**Figure 4:**
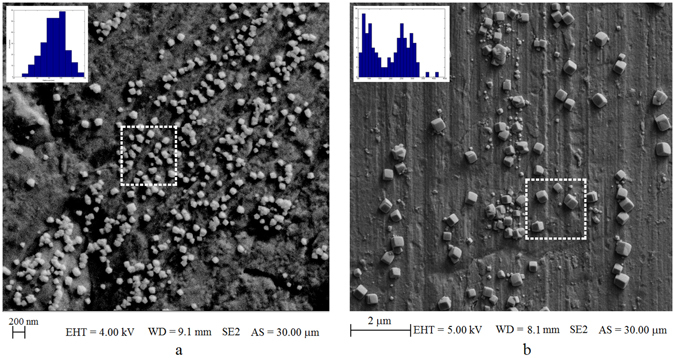
SEM images from anode surface for different electrolysis times when container was filled with 5 *μM* RhB. (**a**) 90 seconds of electrolysis, the inset shows the histogram of nanoparticles in this image. The average is 100 *nm* and the nanocubes size range from 20 *nm* to 150 *nm*. (**b**) 5 minutes of electrolysis time. The inset shows the nanoparticle distribution in this image. There are two peaks, first peak is centered around 100 *nm*, and the second peak is centered around 300 *nm*, and nanocubes size range from 50 *nm* to 400 *nm*. The nanoparticles shown inside the dashed square regions are used in simulations for calculating the electric field distributions and the surface plasmon resonances (see text).

In the next set of experiments nanoparticle production and Raman experiments were performed simultaneously. After replacing the electrodes with a new and clean copper electrodes, we filled the cuvette with rhodamine B (RhB) dissolved in ultra-pure water of concentration 5 *μM*. The conductivity of this solution was 1.56 *μS*/*cm*, and 32 *V* was applied during the electrolysis. SEM images of the nanoparticles formed on the anode after 90 seconds and 5 minutes of electrolysis time are shown in Fig. 4. In this figure, histograms of nanocubes dimensions are shown in the insets. In the 90 second run the average particle size is about 100 *nm* but in the 5 min run there are two peaks centered at 100 *nm* and 300 *nm* of particle size. Although the reasons are unknown, it is interesting that we have two groups of nanocubes differing in size forming on the substrate.

Back scattered Raman signals from the anode surface was recorded as a function of time. Since 5 *μM* solution of RhB is too low of a concentration for a normal Raman spectrum recording it is actually impossible to record the spectrum without SERS enhancement. Therefore, we had to record normal Raman spectrum of much higher concentration RhB solution (100 *mM*) and normalize measurement results to obtain the enhancement factor of our substrate since the Raman intensity is proportional to the number density of the analyte. Namely, to quantify the enhancement of the recorded Raman signal we use^15, 16^:

$$EF=({I}_{SERS}/{I}_{ref})\times ({C}_{ref}/{C}_{SERS}),$$

where *I*_*SERS*_, *I*_*ref*_, *C*_*ref*_ and *C*_*SERS*_ correspond to recorded Raman signal intensity with the SERS substrate, intensity of the reference Raman signal, concentration of the reference sample, and concentration of the SERS sample respectively. Reference recording was performed with a new copper anode before the electrolysis, and after that copper electrode is cleaned and electrolysis is performed with lower RhB concentration. Experiments are performed with utmost attention to prevent left over contamination over the anode. In addition, we also made SERS measurements after replacing the anode altogether that is used for the reference measurements, and the results did not change.

**Figure 5:**
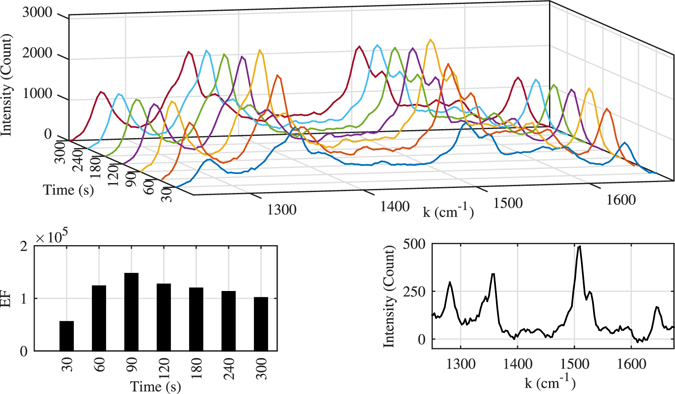
Enhancement factor (*EF*) of 5 *μM* RhB variation for different electrolysis time. For electrolysis time of 90 seconds the *EF* for the Raman peaks of 1509 *cm* ^−1^ reaches the maximum value of above 5 orders of magnitude. All of the spectrums were recorded in one second with laser power of 250 *mW*, and the reference spectrum was recorded with 100 *mM* RhB concentration.

In order to obtain the optimum required time for electrolysis -to find the maximum *EF*- we recorded the Raman spectrum at various electrolysis times. The recorded spectrums from the anode electrode surface and also the variation of *EF* for the 1509 *cm* ^−1^ Raman peak of RhB is shown in Fig. 5. As it is clear from this figure, even for 30 seconds of electrolysis time the anode surface can function as a SERS active substrate. The maximum *EF*, roughly 1.5 × 10^5^, occurred at 90 seconds into the electrolysis then it started to reduce gradually, which after 5 minutes of electrolysis time the *EF* decreased to almost 75% of the maximum.

**Figure 6:**
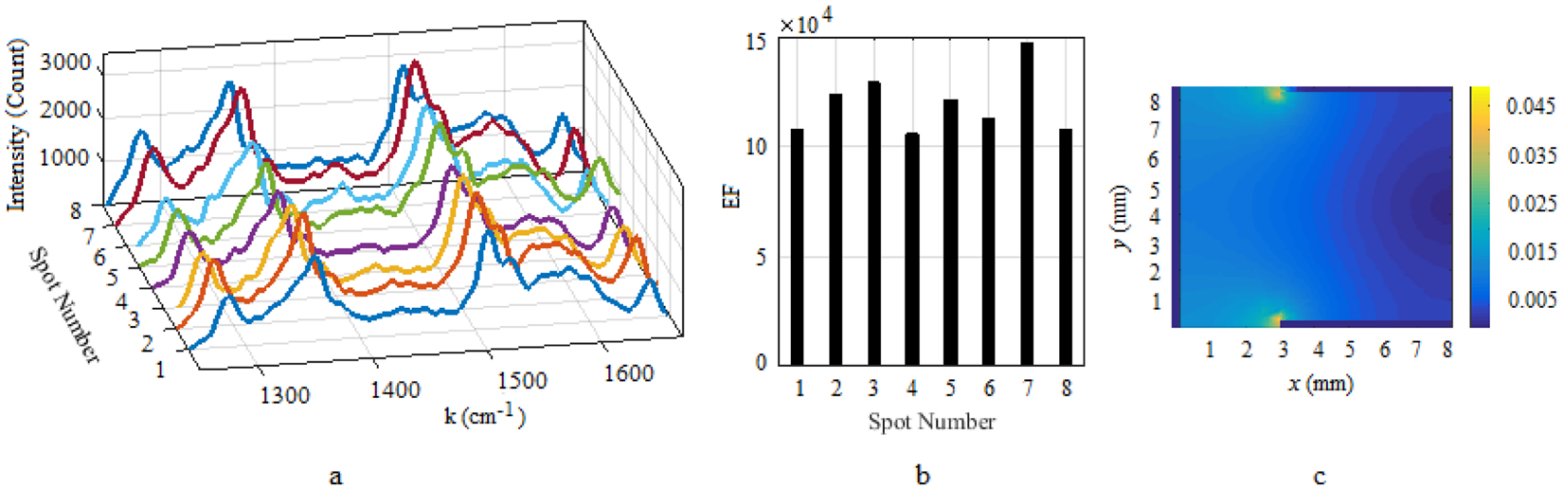
Left: The recorded Raman spectrum from eight random spots on the Cu substrate. Middle: Comparison of *EF* through these spots. Calculated *RSD* of the *EF* variation for the Raman peak of 1509 *cm* ^−1^ is 12 percent. The spectrums were recorded with 90 seconds of electrolysis time and RhB of concentration of 5 *μM*. Right: Simulated current distribution over the cross section of the cuvette. Anode is indicated with solid thick line at at *x* = 0.

This reduction in *EF* can be explained by the change in the dimensions of the nanoparticles on the anode surface. According to captured SEM images (see Fig. 4) from the anode surface, for 90 seconds electrolysis the average size of nanocubes is about 100 *nm*, while by increasing the electrolysis time to 5 minutes, the average size of the larger nanoparticles reaches to about 300 *nm*. Therefore a peak in *EF* is expected, as it will be explained in the discussion section, maximum enhancement should occur for surface plasmon resonance (SPR) wavelengths around 838 *nm* which corresponds to a nanoparticle size of about 100 *nm*. After 5 minutes of electrolysis there is still a group of 100 *nm* nanoparticles left but their number is smaller since some of them grew to larger sizes, therefore a decrease in *EF* is expected. The substrate *EF* uniformity for the Raman peak of 1509 *cm* ^−1^ was checked by measuring the *EF* on eight random spots through a roughly 1 *cm* ^2^ area of the anode surface after 90 seconds electrolysis. The *EF* variation through these spots had a relative standard deviation(RSD) of roughly 12 percent, and the spectrums and the *EF* factors of these spots are shown in Fig. 6. A uniform *EF* was expected from the surface since the current density over the anode surface is rather uniform due to our electrode placement, which leads to uniform nanoparticle production on the anode. Simulated current distribution in the cuvette is also shown in the same figure.

We repeated similar electrolysis experiments with other metal electrodes including brass, Ag, Zn and Al which except brass, no Raman enhancement obtained. Enhancement from brass is understandable since it is an alloy of Cu and Zn. The obtained Raman signal from the brass anode surface is similar to that of copper, while for silver, zinc and aluminum no Raman peak is observed. The electrolysis time for these metals were even increased up to 10 minutes but still no enhancement observed. We have also inspected surfaces of anodes made of Ag, Al, and Zn with SEM and has not observed any nanoparticles. Although the nanoparticles on the anode is responsible for the SERS enhancement, there must be some nanoparticles in the electrolyte as well. However those nanoparticles are expected to be in negligible quantities and too small of size (tens of nanometer in size) as will be discussed later, they would not contribute to the Raman enhancement. For confirmation of this fact we also performed *EF* measurements offline, meaning that RhB solution is applied over the Cu anode electrode after it was removed from the cuvette, and we still obtained similar above five orders of magnitude enhancement of Raman signals.

**Figure 7:**
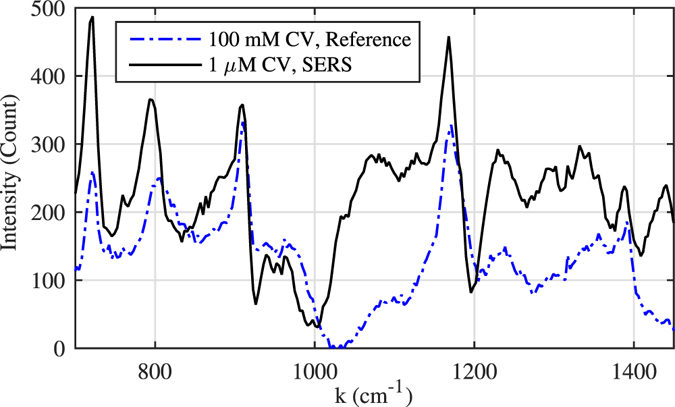
SERS Enhancement of crystal violet (CV) solution: 1 *μM* crystal violet solution on Cu anode after 3 minutes of electrolysis compared to 100 *mM* reference Raman measurement. All recordings were done in 1 sec with 250 mW of laser power. For example, the Raman peak of CV at 1175 *cm* ^−1^ is enhanced for more than 10^5^.

SERS experiments were repeated with solutions of crystal violet (CV) which is widely used as a histological stain, particularly in Gram staining for bacteria classification, and as the Fig. 7 shows the Raman peaks were enhanced again for more than 10^5^ times.

## Discussion

### Characterization of Nanoparticles

**Figure 8:**
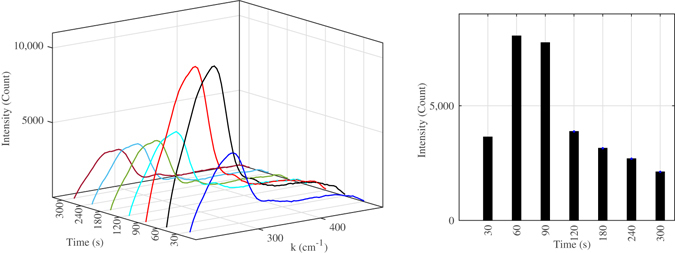
The recorded Raman spectrums from the anode surface at different electrolysis times which show a peak at 290 *cm* ^−1^. The electrolyte was just the ultra-pure water, and the peak intensity changes in a similar pattern as a function of electrolysis time as the Raman measurements done with RhB. This measurement confirms that the nanoparticles are indeed CuO particles.

A traditional way to prove the existence of copper oxide is X-Ray diffractometry (XRD) technique but here we could not use this technique since the nanoparticles are produced on the copper surface, and the amount of signal from the copper plate overwhelms the signals emanating from the nanoparticles. Therefore, in order to determine whether the nanoparticles are copper or copper oxide, following experiment is performed: while the electrolysis is in progress, copper anode was subjected to Raman spectrum measurement. Back scattered Raman spectrum from the copper anode surface was recorded and it clearly showed the Raman peaks of CuO nanoparticles at 290 *cm* ^−1^. Furthermore, this Raman peak intensity changes over the electrolysis time in a similar fashion to the experiment done with RhB experiments which we can conclude that indeed resultant Raman signal is from the forming CuO nanoparticles. Another point to note that CuO maximum peak occurs at 60 seconds electrolysis as shown in Fig. 8, while the maximum *EF* with RhB happens at 90 seconds electrolysis time. This is expected since the Raman peak of CuO at 290 *cm* ^−1^ is closer the laser wavelength than the Raman peak of RhB at 1509 *cm* ^−1^, which means that nanoparticle size should be smaller for the SPR enhancement in this case. The intensity of Raman peak of CuO at 290 *cm* ^−1^ is also decreasing after 90 seconds electrolysis as the case measurement with of RhB indicating that the nanocubes are growing in size.

### Nanoparticle Synthesis

In an electrochemical cell, when a voltage is applied between the anode and cathode, electrons are removed from the anode and they are replaced from the cathode. Metal ions are extracted from the anode (cations) and migrate to cathode where the reduction takes place and metallic clusters form. When a metal salt is used in the electrolyte such as copper sulphate (CuSO_4_) in the case of copper nanoparticle production with copper anode, due to ionization, CuSO_4_ is dissociated into Cu^2+^ and ${{\rm{SO}}}_{4}^{2-}$ ions. The current is carried by Cu^2+^ and ${{\rm{SO}}}_{4}^{2-}$ in the solution, and Cu^2+^ ions move towards the cathode. Some of those cations get reduced in the electrolyte, the remaining ones arrive to cathode and they get reduced to Cu atoms and deposited on the cathode. The metallic clusters are formed over time and this process takes hours to days and the size of nanoparticles produced are on the order of tens of nanometers to hundred nanometers range^23, 24^. Therefore in matter of minutes the size of nanoparticles deposited on the cathode would be too small to be suitable for SERS applications as the surface plasmon resonance frequencies would be towards the UV range and would not be applicable for Raman spectrum measurements.

In contrast to those studies, when we performed electrolysis with copper anode in ultra-pure water we observed copper oxide nanoparticles over the anode surface in matter of minutes at the sizes of up-to 400 *nm*. When the anode material is copper we contemplate the reactions listed in Table 1 to occur on the surface.

**Table 1:** Reactions on the anode.

H_2_O → H^+^ + OH^*−*^
Cu → Cu^2+^ + 2*e* ^*−*1^
2H^+^ + 2*e* ^*−*1^ → H_2_
Cu^2+^ + 2OH^*−*^ → CuO + H_2_ *O*

Some of the oxidized copper atoms remain attached to surface and combine with OH^−^ to form Cu*O* and start the crystallization to form the nanoparticle in cubic form. There should be copper cations moving towards the cathode and reduced on the way, or at the cathode but their cluster size must be negligible in size because of the short of amount of electrolysis time. When the experiments are repeated for anodes made of other metals such as brass, Al, Zn, and Ag, except brass, no nanoparticles on the anode is observed during the electrolysis times for up-to ten minutes. The cations from the anode are in competition with *H* ^+^ to gain the electrons, hence for the reduction to form the relevant atoms or their oxides on the anode, and the electrode potential will determine which cation will win the competition.

According to electrode potentials listed in the Table 2, copper cations have much more tendency to get reduced compared to hydrogen (*H* ^+^), therefore, more copper cations will gain electrons in competition with *H* ^+^. However, considering the aluminum and zinc cations, hydrogen has more reduction tendency in comparison, hence, they will fail to gain electrons and get reduced to produce enough metal atoms and/or metal oxide nanoparticles especially in such short electrolysis times. There is an interesting point to note when the anode electrode is made of brass. Since brass is composed of Cu and Zn elements with ratio of approximately 70/30, at first glance it is not surprising to have CuO nanoparticle formation. However on the other hand, the oxidation potential of Zn is more electropositive so that one can expect ZnO formation instead of CuO. In reality, Zn loses electrons easier than Cu (hence oxidizes faster), however in order ZnO or CuO to form, Zn and Cu ions have to be reduced. In that case Cu ions wins the competition (since oxidation and reduction potentials are opposite of each other). Besides Cu ions, electron capture rate of *H* ^+^ is also higher than Zn ions. Therefore, as we indicated above, when *H*_2_ *O* forms *H* ^+^ and *OH* ^−^, the oxygen from *OH* ^−^ reduces the Cu ions to form CuO. Then Zn ions must travel toward the cathode and they get reduced there. The Raman spectrum of the copper anode and brass anode both show the CuO Raman peak (at 290 *cm* ^−1^) but the brass anode does not show any of ZnO Raman peaks (for example, 434 *cm* ^−1^ and 500 *cm* ^−1^). Also in a separate experiment, nanoparticles falled out from the brass anode are collected on a stainles-steel plate. The EDS analysis of this plate shows that Cu and O elements are present at significant amounts (besides the elements present in the steel) but the amount of zinc is negligible.

**Table 2:** Reduction potentials of the metals.

Reaction	Electrode Potential (V)
*Al* ^3+^ + 3*e* ^−^ → *Al*	−1.66 V
*Zn* ^2+^ + 2*e* ^−^ → *Zn*	−0.76 V
2*H* ^+^ + 2*e* ^−^ → *H*_2_	0.00 V
*Cu* ^2+^ + *e* ^−^ → *Cu* ^*+*^	0.15 V
*Cu* ^2+^ + 2*e* ^−^ → *Cu*	0.34 V
*Ag* ^+^ + 1*e* ^*−*^ → *Ag*	0.8 V

For the silver, electrode potential is much bigger than that of the hydrogen, hence much more silver atoms and consequently nanoparticles (compared to copper) were produced inside the liquid during the experiment but the lower tendency of silver for oxidation limits silver oxide nanoparticle growth on the anode surface in short time. Also, the silver nanoparticles in the electrolyte must be in too small of a size and/or small number of quantity to contribute to the real-time Raman signal enhancement in experiments performed, especially for our short electrolysis durations. We confirmed this view by analyzing the dried out electrolyte -after 10 minutes of electrolysis- with EDS and XRD methods. EDS measurements indicate majority of nanoparticles are indeed silver with a neglibible amount of oxygen presence. XRD analysis indicated that silver nanoparticle dimensions are in the range of 10 to 15 nm.

### Surface Plasmon Resonance Simulations

**Figure 9:**
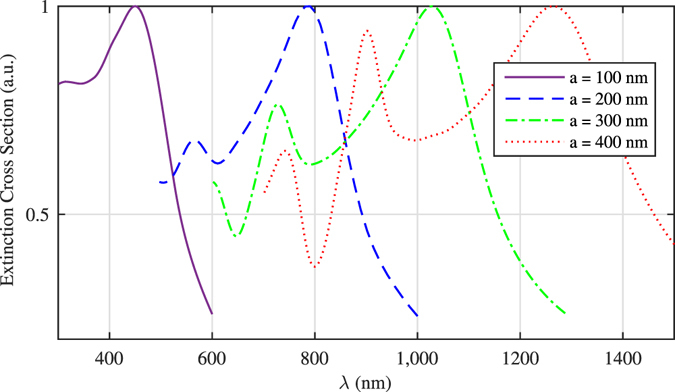
Simulated SPR of copper oxide nanocubes of different size; increasing the nanocubes size leads to red shift in SPR wavelength and also excitation of the higher order resonance modes.

As far as the SERS phenomenon is concerned usually noble metals and transition metals are used for the SERS substrates. However metal oxides are also confirmed to act as the SERS active substrate^15^. The first experiment on the use of metal oxides as the SERS substrate was reported in 1983^33^, and this area is still research in progress. There is also a report showing the capability of copper oxide as a SERS active substrate with enhancement factor of two orders of magnitude^34^, and also some late work on metal oxide SERS-active substrates by defect engineering^35^. In general it is contemplated that there is a charge transfer effect within the metal/metal oxide interface. The charge transfer allows manipulation of the localized surface Plasmon resonance and finally the Raman signal enhancement factor^36^. In our case, we performed our simulations for copper oxide nanoparticles over a metallic surface therefore this effect is included. When the simulations are performed without metal backing there is only semiconductor/dielectric resonances of the particles and the resultant electric fields are not as high as when the copper oxide nanoparticles sitting over copper surface. The effect of nanoparticle size on the *EF* factor is investigated using finite difference time domain (FDTD) technique by calculating the surface plasmon resonance (SPR) wavelength of nanoparticles. The simulation is performed first for a single nanocube of size ranging from 100 *nm* to 400 *nm*, with cubic mesh and minimum mesh step size of 4 *nm* and the time step size stability factor of 0.85. The calculated extinction cross sections of the nanocubes as a function of wavelength is shown in Fig. 9 below.

**Figure 10:**
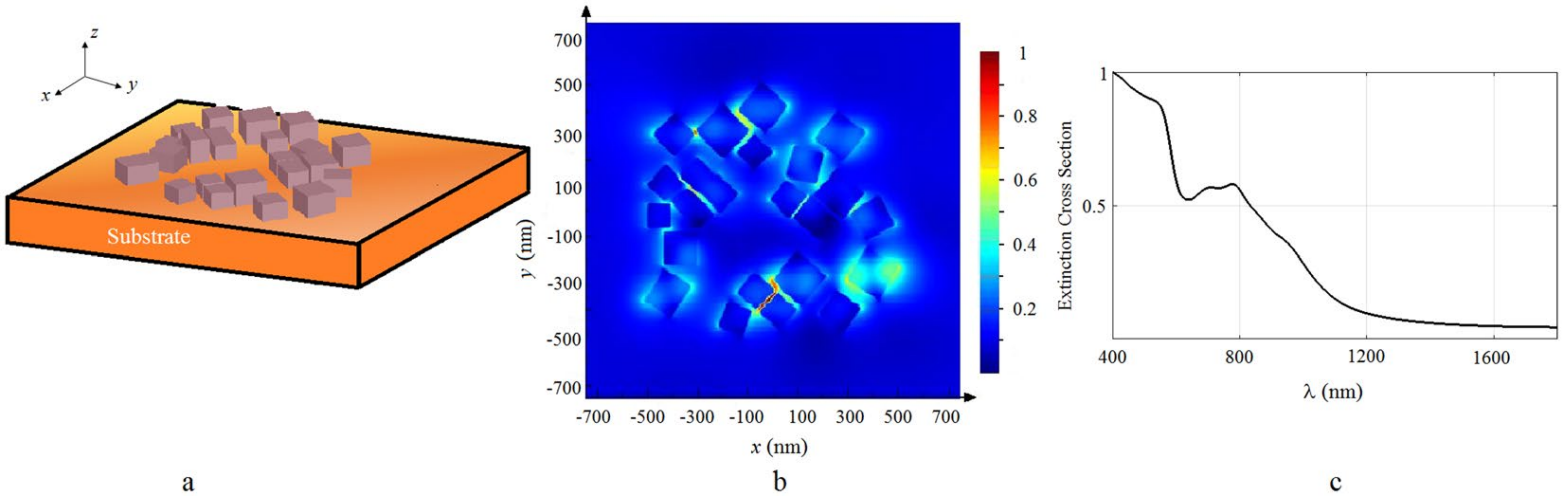
(**a**) The simulated area is 1 *μm* × 1 *μm* where size of copper oxide nanocubes range from 85 nm up to 150 nm, inter-particle gaps range from 10 *nm* to 350 *nm*. (**b**) Electric field amplitude distribution at 50 *nm* above the surface at SPR resonance of 794 *nm*. (**c**) The extinction cross section of this assembly of nanocubes as a function of wavelength.

As it is clear form this figure the growth in nanocubes size leads to red shift in surface plasmon resonance peaks and also produce other SPR resonance wavelengths -due to excitation of higher-order resonance modes- appearing at shorter wavelengths. The red shift in SPR causes a mismatching between the SPR peak and the excitation source wavelength used, which is the reason for *EF* having a maximum as a function of electrolysis time. However the single nanoparticle simulation is not really reflecting the reality since it does not include the mutual coupling effects between the nanoparticles and the substrate. Light intensity should increase in the gap between the nanoparticles and on the sharp corners and crevices, therefore a more realistic simulation is in order for calculating the field distribution and the extinction cross section. For this purpose we selected a realistic collection of particles as shown with the dashed squares in Fig. 4. For the ninety second run simulations, we selected the 1 *μm* × 1 *μm* area shown in this figure and performed a FDTD simulations. Here we assumed nanocubes are copper oxide nanocubes of size ranging from 85 *nm* to 150 *nm* in accord with the particle dimensions on that selected square. Inter-particle distances were assumed to range from 10 *nm* up to 350 *nm* and the nanocubes were sitting on copper substrate. The electric field was assumed to propagate perpendicular to the substrate surface. The values for the refractive index, n, and the extinction coefficient, k for copper and copper oxide are taken from literature^37, 38^. The results of this simulation is shown in Fig. 10, where the particle distribution, electric field distribution at the surface plasmon resonance of 794 *nm*, and the extinction cross section as a function of wavelength is shown.

**Figure 11:**
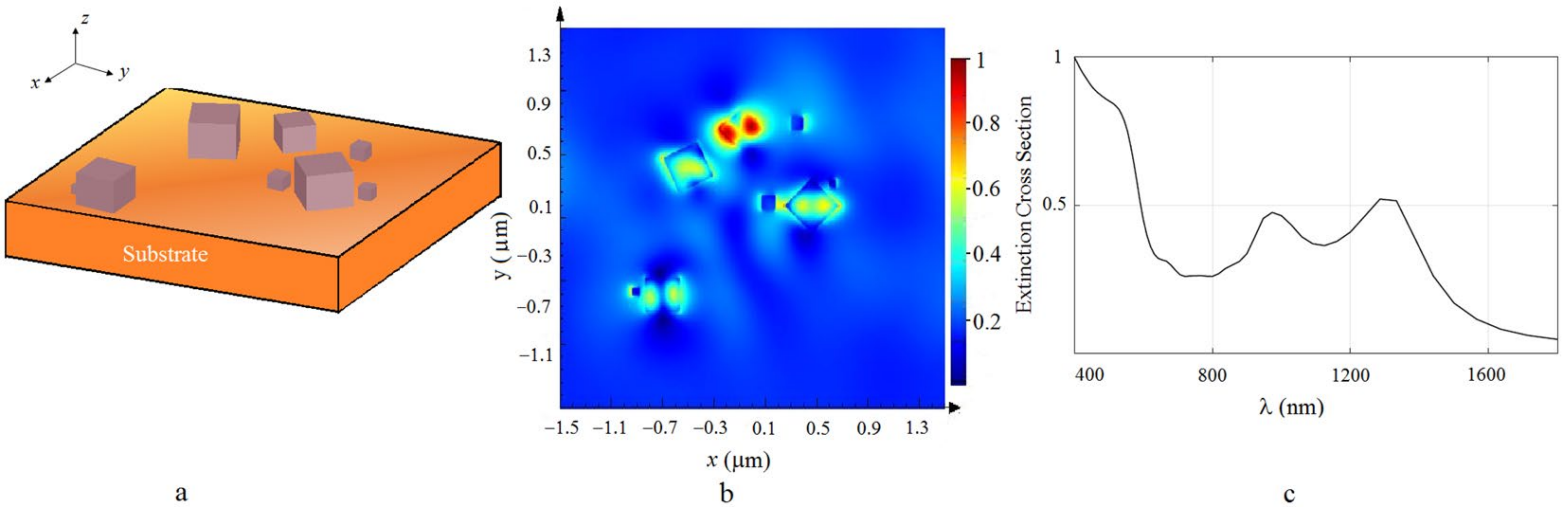
(**a**) The simulated area is 2 *μm* × 2 *μm* where size of copper oxide nanocubes range from 60 *nm* up to 300 *nm*, inter-particle gaps range from 50 *nm* to 1000 *nm*. (**b**) Electric field amplitude distribution at 50 *nm* above the surface at SPR resonance of 971 *nm*. (**c**) The extinction cross section of this assembly of nanocubes as a function of wavelength.

Same simulation is repeated for larger particle distribution which is obtained by five minutes of electrolysis. In this case The simulated area was 2 *μm* × 2 *μm*, and the nanocube dimensions were ranging from 60 *nm* to 300 *nm* with inter particle gaps ranging from 50 *nm* to 1000 *nm* as estimated from Fig. 4. The results of this simulation is shown in Fig. 11, where the particle distribution, electric field distribution at the surface plasmon resonance of 971 *nm*, and the extinction cross section as a function of wavelength is shown.

Although, according to theory the maximum SERS enhancement occurs when the substrate resonates at laser wavelength, in practice the maximum enhancement takes place when the SPR resonance occurs around the middle of the excitation wavelength and Raman scattering wavelength^39^. Since the laser source has a wavelength of 785 *nm*, the Raman peak of RhB at 1509 *cm* ^−1^ (corresponds to 891 *nm* in terms of wavelength), therefore we would expect enhancement for SPR resonances around 838 *nm*. According to our simulations for a typical distribution of nanoparticles at 90 seconds, the resonance wavelength is around 794 *nm* for average nanoparticle size of 100 *nm* as shown in Fig. 10. Considering that the particles are distributed randomly and the selected area produces resonances around the expected wavelengths, we can conclude that the experimental results are in good agreement with the theoretical predictions; as the SEM images in Fig. 4 show, the average size of nanocubes are about 100 *nm* when the maximum *EF* occurs. When the electrolysis time is extended to 5 minutes the resonant wavelength is shifted to 971 *nm* as observed from Fig. 11. Also it is known that as the nanoparticle size grow comparable to the wavelength of excitation, the nonradiative modes are excited on the particles which diminishes the SERS enhancement factor^40^. We might also see this effect come into play for longer electrolysis times.

## Conclusion

In conclusion, we have experimentally found that using just the ultra-pure water as the electrolyte and the copper electrodes, ions extracted from the anode form copper oxide nanoparticles on the anode surface in matter of minutes. As a proof of principle experiment, we used this anode electrode as a SERS substrate for enhancing the Raman peaks of RhB and CV and obtained *EF* factors over five orders of magnitude. The proposed method has some key advantages over existing SERS substrates: it is not only a real time SERS substrate but also is a very fast, simple and a low cost technique. This technique also allows preparation of a large effective area for SERS enhancement, virtually unlimited area as long as the current is uniform over the substrate during the electrolysis. Although nanocubes are the only nanoparticles produced and in random orientation, their size is controllable, and the repeatability of the substrate, as far as the nanoparticle size is concerned, is excellent. Furthermore this substrate is tunable in wavelength, although irreversibly and only in one direction. We also investigated other metal electrodes including Ag, Al, Zn and brass for their real time SERS substrate capability, which none of them except brass exposed this capability, nanoparticles were produced only at copper and brass anodes for electrolysis times up-to ten minutes. An explanation for CuO nanoparticle formation on anodes made of copper and brass but not with other metals is given using standard electrode potentials of the metals. Also, surface plasmon resonance simulations of the nanoparticles were performed in accord with the nanoparticle distributions obtained from the SEM images. The simulation results agree with the change in SERS intensity as a function of time since as the nanoparticles grow in size SPR wavelengths shift.
